# Dental Progress. No. 5

**Published:** 1868-03

**Authors:** 


### ARTICLE III.

### *Dental Progress. No.* 5.

By Professor Austen.

A good "impression," the basis of a dental mechanism, is the resultant of (1) skill, applying a (2) good plastic material, by means of (3) a suitable vehicle. In considering these three, in connection with dental progress—we have already shown the advantages possessed by the operator of the present day, in respect of the last; and we propose, in this paper, to show the same, in respect of the second. But in the first we can claim no progress.

The dentist of to-day is not a whit more skilful than the dentist of the last century—taking a fair representative from each age. The piece before me, made by Hudson, shows evidence of a skill equal to that displayed by a modern *chef d'œuvre*, although very inferior in point of beauty, utility and durability. Skill, talent, genius exist the same in all ages. But the *results* of these, applied to science or mechanism, will vary in direct proportion to the material and instruments used. Thus Herschel and Rosse unfold to us more of the heavens than the superior genius of Galileo or Copernicus could do: and La Place with his improved calculus solved problems, that defied the wonderful powers of the great Newton. Were Archimedes living now, his steam engine would be the world's wonder. As his genius, acknowledging nothing impracticable, asked only a "place to stand" that he might "move the world:" so mechanical genius, in all ages, ask only the material and the tools for working out the "impossible."

Let us then be careful that in the pride of progress, we do not under-value the genius of the past. Perhaps with our means, it would have done more than we: certain it is, that for what we can do, we are largely indebted to what it did before us. The genius of an era is to be measured less by results attained, than by difficulties overcome. Indeed, it is not a little to be feared that the abundance of resource, in certain departments of dental art, has induced a neglect of skill.

Applying these thoughts to the practical points now under consideration—we cannot take any better impression than did Hudson, Greenwood or Gardette unless we have better materials and appliances; and not so good, with all these advantages, unless we exercise more or less of their skill. The cup used in those days was suited to the work then made. As the style of work improved, inventive talent supplied a corresponding improvement in the forms of impression cups.*

Change in the styles of work demanded also change in the impression material. When plates were made of bone,

---

*Since our last article, we are pleased to notice that an enterprising firm have supplied the profession with the form of cup (the outer curve meeting the flat bottom of the cup at a right angle) for partial impressions, there referred to, and which we regard as an essential part of a dental outfit.

We should have mentioned also, among the improvements in this department, Dr. Bean's very simple and ingenious method of making the "swaged cup" adhesive on its palatine surface, so as to retain very thin layers of plaster.

ivory or hippopotamus tooth, the minute accuracy of plaster would have been useless, since the carving out of the plate and fitting by "trial" could give only approximate results. So also, when the French artificer takes his lump of gold and, with his hammer and stake, works out his plate and fits it by "trial," the same holds good. Also in case of the porcelain plate which, becoming too small by shrinkage, is ground out by wheels and fitted by "trial," an exact fit is impossible.

But when we use a plastic material, which like the vulcanite, copies the minutest lines, it is clear that we require an impression material capable of more accurate definition than wax can give. Yet even here, without inquiry into the characteristics of the vulcanite plate, we cannot decide upon the relative merits of plaster and wax.

The introduction of plaster and gutta-percha as impression materials is undoubtedly a very great improvement; for with them we can do in a large class of cases what, otherwise would be impossible, or else imperfectly done. But when we adopt either of these valuable materials, to the exclusion of wax, we rob our laboratory of a material as useful as it is old-fashioned. It is scarcely too much to affirm that the exclusive use of plaster is no improvement upon the exclusive use of wax: any more than the introduction of plastic work justifies the exclusion of swaged work.

True progress lies in "abundance of resource," from which may be drawn what is best suited to special cases. And unless one inquires carefully into the distinctive properties of a given material, and also into the distinctive wants of a given style of work, it is impossible for him to become a progressive practitioner. Without this discrimination the advocates of wax and plaster may debate *ad infinitum* and still be like the knights of the silver and golden shield—easily reconciled by a glance at the other side. Briefly, wax can do what plaster cannot and plaster can do what wax cannot, and either will do very well, all that ordinary operators seek to accomplish.

Now let us apply these principles, in deciding how far we have improved upon the one impression material of the older dentists—bees-wax.

It becomes by heat very soft and plastic, but rather less so than gutta percha and much less so than plaster. Hence soft parts that must be copied *in situ* require plaster, which permits the softest tissues to preserve their normal relation. But, for the same reason, wax is best in all those cases—and they are not few—in which the membrane covering the alveolar ridge and palate is to be copied in its state of closest compression. The careful operator need not be told that there are cases, in which it is difficult to decide which plate will prove more serviceable, that fitting the loose, or that fitting the compressed, tissue: but this difficulty does not affect the present question, and plaster alone cannot meet all cases.

Wax is absolutely non-elastic and does not contract, on cooling. In these respects it differs from gutta-percha which, especially if too cool, offers an elastic resistance under pressure, and which has a very decided contraction. The routine-practitioner rejects gutta-percha because of this last-named property; unless he can control it, by causing the material to cling fast to the cup (making it adhesive by a momentary application of dry heat before placing the plastic mass in the cup). The eclectic operator recognizes, in this property, an advantage possessed by no other impression material and allows it full play. Hence he will succeed with vulcanite plates, in a certain class of cases, where plaster will certainly, and wax probably fail.

We have just said that wax does not contract. Many persons, misled by over-exact experimenters, hasten to pour their plaster models, while the impression is still warm—a wholly unnecessary haste. Possibly the contraction of *melted* wax misleads many. The contraction of a lump of plastic wax, in which the semi-crystalization is completely broken up by the necessary kneading, is a very different thing. Theoretically it is very slight, practically it is nothing; in any case it is more than offset by the expansion of the model.

This property of wax places it midway between gutta-percha with its marked contraction and plaster with its expansion. Now it is an unquestionable advance in dentistry, which gives this choice of three materials that shall form a model—smaller than, equal to, or larger than, the mouth. For, paradoxical as it may seem, we utterly deny that an "exact fit" is always the best fit. So long as mucus membranes differ in texture, so long is it essential to discriminate between plates that must exactly fit and those which should be smaller—and if smaller, whether uniformly or irregularly so.

It will be seen therefore that the expansion of plaster may be an objection to its use. The objection lies with much force in certain cases: but becomes unreasonable unless we carefully inquire into; *first*, the actual effect of this expansive property on the impression itself: *secondly*, the kind of mouth to be fitted: *thirdly*, the class of work to be made.

As to the first point, the simple statement of expansion will not explain the differences in the four following plaster impressions of the same mouth—first, with a thick layer of plaster in an ordinary rigid smooth brittania cup: second, the same in a soft and rather flexible cup; third, a very thin layer in a cup swaged for the case, or in a strong gutta-percha cup; fourth, a thin layer, in one case adherent to a rigid cup and in the other case free to expand upon it. These differences cannot be appreciated, (for it is almost impossible to measure them unless by their practical result upon the fit of the plate,) except by careful inquiry into the amount and force of expansion. If, like the expansion of metal, it is irresistible, then any attempt to restrain it will be apt to result in a warping of the impression, fatal to success. If it can be controlled by the cup, the manner and amount of force necessary must be considered. Again, if the expansion is desirable, care must be taken that it is not prevented. Thus we see that plaster impressions are not necessarily uniform. Important practical differences will result from the method employed, and we shall have—one, a warped impression, one slightly larger, and a third decidedly larger than the mouth. A series of careful experiments would add much to our knowledge of the exact shape of plaster impressions, and might assist materially in accounting for the failure of some plates, made from plaster impressions, inexplicable on ordinary grounds.

For the various forms of plastic work, such as vulcanite, cast aluminum and cheoplasty, applied to *partial* cases, impressions in plaster are essential to accuracy. For partial vulcanite cases, neither wax nor gutta-percha are at all comparable with it. Whereas for *full* vulcanite cases, wax is in many cases much to be preferred and sometimes the shrinkage of a gutta-percha impression will give an excellent adaptation. These remarks apply to the upper jaw; in the lower jaw, plaster is inferior to either wax or gutta-percha, whatever may be the style of work, unless the ridge is firm and well defined.

Cast aluminum, owing to its great shrinkage, demands all possible compensation, that the expansion of plaster can give; hence, for this work, all full sets should be taken in plaster. The same is true of tin and other cheoplastic metals; but in less degree, as the shrinkage is much less.

Swaged plates made upon zinc dies do not permit the use of gutta-percha, unless shrinkage can be prevented. The contraction of the zinc gives all the reduction in size which some mouths require, and more than others will allow. Hence plaster impressions for full cases are proper here, when they might not answer for the same cases in vulcanite. The advantage frequently urged in favor of plaster—the minute accuracy of the fine lines and rugæ—holds good in plastic work; but in swaged work it amounts to nothing, for zinc (the hardest metal used for dies) cannot impress these fine markings upon a gold plate ; nor are they necessary.

A decided advantage of plaster is the permanence of its shape under the strain of removal from the mouth. Much skill is required to prevent the distortion of wax and gutta-percha, and they should be made as hard as possible by cold cloths, before removal is attempted. But a novice, attempting a partial impression with gutta-percha, when the inclination of two teeth forms a dove-tailed space, and making it *very* hard before attempting to withdraw it, will learn a lesson not soon forgotten. In such cases wax will yield at the expense of accuracy ; plaster will break and the pieces can be replaced ; but gutta-percha neither gives nor breaks.

For use in cold weather paraffine improves the plastic quality of wax, causes it to soften at a lower heat and to become harder when cooled in the mouth. For winter use we consider it by far the best form of wax in use : but quite inadmissable in warm weather unless it is hardened in the mouth with ice. For summer use the older dentists employed white wax or certain forms of vegetable wax. The compound of wax and gutta-percha is well adapted to the same end : but its color is not very agreeable to fastidious patients.

In conclusion we may thus sum up the progress of dental art in the matter of impression materials. We have in addition to the one material of the older dentists, WAX, two valuable modifications thereof, by mixture with paraffine and with gutta-percha : and two new and most important materials, PLASTER and GUTTA-PERCHA. Having certain plastic properties in common, they have distinctive qualities, which when correctly understood, add very largely to the dentist's ability to meet the requirements of new styles of work, unknown fifty years ago. The dentist of to-day can afford to neglect none of the three : and the young dentist, who neglects wax because it is oldfashioned is as ignorant of what constitutes *real progress* as the old dentist, who will not adopt the newer materials. The important work of taking impressions may, not inaptly, be, likened to a tripod: any one of its three feet taken away, it has an uncertain support.

### SELECTED ARTICLES.

